# *Circumnutation Tracker*: novel software for investigation of circumnutation

**DOI:** 10.1186/1746-4811-10-24

**Published:** 2014-07-25

**Authors:** Maria Stolarz, Maciej Żuk, Elżbieta Król, Halina Dziubińska

**Affiliations:** 1 Department of Biophysics, Institute of Biology and Biochemistry, Maria Curie-Skłodowska University, Akademicka 19, Lublin 20-033, Poland

**Keywords:** Circumnutation, Time-lapse, Plant movement, Software, *Helianthus annuus*

## Abstract

**Background:**

An endogenous, helical plant organ movement named circumnutation is ubiquitous in the plant kingdom. Plant shoots, stems, tendrils, leaves, and roots commonly circumnutate but their appearance is still poorly described. To support such investigations, novel software *Circumnutation Tracker* (*CT*) for spatial-temporal analysis of circumnutation has been developed.

**Results:**

*CT* works on time-lapse video and collected circumnutation parameters: period, length, rate, shape, angle, and clockwise- and counterclockwise directions. The *CT* combines a filtering algorithm with a graph-based method to describe the parameters of circumnutation. The parameters of circumnutation of *Helianthus annuus* hypocotyls and the relationship between cotyledon arrangement and circumnutation geometry are presented here to demonstrate the *CT* options.

**Conclusions:**

We have established that *CT* facilitates and accelerates analysis of circumnutation. In combination with the physiological, molecular, and genetic methods, this software may be a powerful tool also for investigations of gravitropism, biological clock, and membrane transport, i.e. processes involved in the mechanism of circumnutation.

## Background

Circumnutations have been investigated for over 130 years [1-4] but the methods for analysis thereof are still time consuming and weakly standardized. During his investigations, Charles Darwin recorded circumnutations in many plant species simply using a glass plate [1,4-6]. More recently, circumnutations have been investigated using a photo-diode containing apparatus [7] and currently by time-lapse images [8-10]. Some investigations search the core mechanism [11-13] and ultradian pacemaker of circumnutation [14]. The circumnutation analysis also accompanies investigations of gravitropic responses [10,15,16], growth mechanisms [17-20], ethylene signalling [21], IP_3_ signalling [22], and glutamate signalling pathways [23] as well as investigations of aluminium treatment [9] and circadian clock [24-26]. Other studies explore the function of circumnutation in plant life [27-29]. Circumnutation analyses also accompany the research of right- and left-handed symmetry of twining organs [6,30] and they should be taken into account in investigations of heliotropism of organs [31]. The geometry of circumnutation is dependent on the morphological traits of plants [7,32,33]. Software for tracking root growth and development [34-36] or measuring hypocotyls and leaf rosettes [37,38] are known, but there is no tool for analysis of circumnutation – a ubiquitous phenomenon in plants. The aim of our work was to design software for analysis of standard circumnutation parameters in relation to the geographical direction applicable in various plant species. To our knowledge, the *Circumnutation Tracker* (*CT*) software presented here is the first free and open source tool for analysis of circumnutation.

### Implementation

The *CT* is based upon cross-platform solutions and runs under the Windows (XP, Vista, 7) and Linux environment. Automatic options use Basic Linear Algebra Subroutine (BLAS) for calculations. The following steps are necessary for software installation: download *Circumnutation Tracker* and *CT* user guide (pdf) to your computer from http://circumnutation.umcs.lublin.pl and run: Circumnutation Tracker.exe. All details of work with *CT* are presented in the *CT* user guide.

### The CT workflow

**Figure 1:**
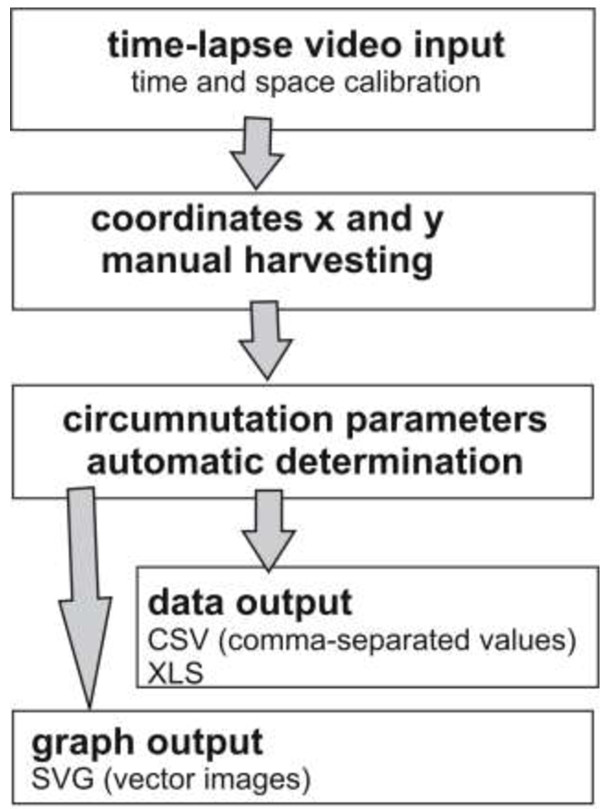
**The workflow with *Circumnutation Tracker.***

The scheme of workflow with *CT* is presented in Figure 1. The crucial steps of working with *CT* include time-lapse video loading, calibration of time and space, manual harvesting of coordinates x and y, automatic determination of circumnutation parameters, and data and graph exporting.

### Time-lapse video input - parameters of time-lapse video files opened by CT

*CT* works on black/white or colour time-lapse video images (AVI Audio Video Interleave), with any resolution. The supporting video codec’s include H.264 and Indeo Video 5.

### Time and space calibration

Circumnutating plants are elaborated by *CT* on a time-lapse video and circumnutation parameters are expressed in minutes and millimetres. The organ apex is usually chosen for tracing the circumnutations and their coordinates x and y changing during the experiment are determined. The centre of the coordinated system can be set to a preferred location e.g. in a place such as organ origin, by the *setup origin* option. Coordinates x and y harvested from the time-lapse video can be previously calibrated by the *frame timestamp* and *perspective correction* options. All technical details of work with *CT* are described in *CT* user guide.pdf.

### Timestamp

Coordinates x and y are labelled with time stamps: year, month, hour, minutes, and seconds by the *frame timestamp* option.

### Calibration and perspective correction

Conversion of pixels to millimetres and elimination of the illusion of an enlarged image when the plant grows toward the camera are performed in the *perspective correction* option using: frame width at the ground level in a pot, distance of the camera from the ground, plant height at the start and end of the experiment. Plant height at the end of our experiment was different for distilled-water and nutrient-solution growing seedlings (Table 1). Thus, the calibration and perspective corrections were made in different database files: Sample 1 (distilled water.db) and Sample 2 (nutrient solution.db), downloaded with the *CT* software.

**Table 1:** **Comparison of circumnutation parameters of *Helianthus annuus *** **seedlings growing in distilled water and a nutrient solution**

**Seedling numero**	**Hypocotyl length (mm)**	**Period (min)**	**Length (mm)**	**Rate (mm min**^**-1**^**)**	**Shape**	**Angle (°)**	**cw%**	**ccw%**	**?%**
D i s t i l l e d w a t e r									
1	85	270	18	0.07	0.43	-24	0	69	31
2	60	267	16	0.06	0.21	1	0	33	67
3	80	335	36	0.12	0.31	-3	40	30	30
4	90	293	19	0.07	0.21	-15	11	22	67
5	70	195	9	0.05	0.44	24	0	0	100
6	75	275	9	0.04	0.38	-43	8	0	92
7	65	400	11	0.03	0.54	-36	0	0	100
8	80	260	6	0.02	0.51	-20	0	0	100
Mean	76*****	287**	15**	0.06	0.38*	-15	7	19	73
SE	4	21	3	0.01	0.04	8	5	9	11
N u t r i e n t s o l u t i o n									
9	115	225	28	0.11	0.59	0	0	53	47
10	105	189	25	0.12	0.35	-17	21	16	63
11	112	188	97	0.48	0.57	3	11	74	16
12	116	217	42	0.19	0.61	-33	81	6	13
13	116	218	48	0.21	0.40	-40	69	6	25
14	113	175	73	0.40	0.56	-11	10	86	5
15	111	225	47	0.21	0.33	-30	6	81	13
16	121	209	99	0.47	0.55	6	76	18	6
Mean	114	206	57	0.27	0.49	-15	34	42	23
SE	2	7	10	0.05	0.04	6	12	12	7

The results were estimated for significance by a t-test at p < 0.05 *, p < 0.01 **, p < 0.00001 *****.

### Geographical directions

**Figure 2:**
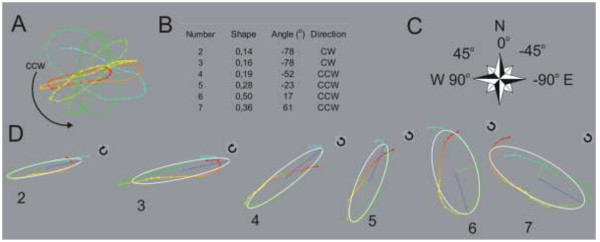
**Fitting the shape, angle, and direction of circumnutations. A**. The top view of the trajectory of circumnutation of the hypocotyls of one-week old *Helianthus annuus*. The example of seedling number 14 on Additional file 1: Video 1. The beginning and end of the circumnutation trajectory are marked by colour gradients from red to blue line **B**. Shape, angle, and direction of circumnutations number 2–7 (also in a Table 2). **C**. Arrangement of circumnutation angles in the geographical direction plane. **D**. Subsequent circumnutations with a marked shape (ellipse, white line), angle (blue line), and direction of circumnutations. In the geographic direction plane, the single circumnutation cycle is determined by two subsequent maximum northward bends of the hypocotyl.

In many papers, the circumnutation trajectories are presented as viewed from above [15,18,28,39]. For a better understanding of the geometry of circumnutation in relation to plant morphology, we propose to set a top-view camera corresponding to the geographical plane and thus the coordinates x, y simultaneously correspond to the geographical east–west (EW) and north–south (NS), respectively. The seeds, seedlings, or older plants can be set to the experiment by juxtaposing their symmetry to the NS-EW directions (Additional file 1: Video 1, Figure 2C). We have an unquestionable point of reference for the circumnutation trajectory (especially circumnutation shape, angle, and direction) to the morphology of plants and environmental geographical directions by such settings of equipment and plants. This could also contribute to future studies on the role of circumnutation in morphogenesis including a study of right- and left-handing and symmetry of development. Additionally, linking the camera settings and plant symmetry with geographical directions will be useful for studying circumnutation together with heliotropism and phototropism [31]. The benefit of *CT* is that we can study every single circumnutation in an objective NS-EW plane, which is an advantage over Fourier and autocorrelation analysis as these provide information about frequency only.

### Coordinates x and y are manually harvested

**Figure 3:**
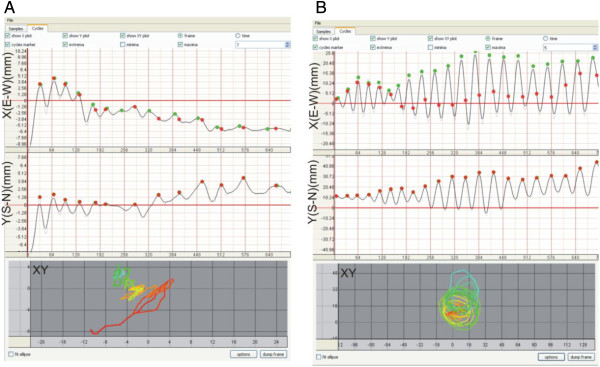
**Screen shoots of the panel for determination of circumnutation cycles.** Regular and irregular pattern of circumnutation can be elaborated successfully by *CT*. **A**. irregular (seedling 6 in Additional file 1: Video 1 and Figure 4A) and **B**. regular pattern (seedling 14 in Additional file 1: Video 1 and Figure 4B) of trajectory of circumnutation of *Helianthus annuus* hypocotyls lasting 63 hours. The black line is smoothed by a moving average filter; the green dots are maximum extremes calculated from coordinates x and y, the red dots are extremes confirmed by the user and its mark a single circumnutation between two maximum northward bends (more info in *CT* user guide.pdf).

Manual harvesting of the organ apex coordinates x and y by computer mouse clicks is controlled by the user. The coordinates x and y can be exported as a CSV (comma-separated values) file that can be opened in spreadsheet software (e.g. Microsoft Excel). The coordinates x and y presented as a time series are shown in Figure 3 for a *Helianthus annuus* hypocotyls. The manual harvesting is an advantage due to the independence of the background of circumnutating plants; therefore, *CT* is applicable in a wide range of filming conditions. We are also working on development of automatic harvesting of coordinates x and y as a prospective *CT* option.

### Circumnutation cycles and parameters are automatically determined

#### Calculation of the extremes

After harvesting, the graphs of the time series of coordinates x and y are automatically plotted and an algorithm for calculation of extremes starts running (Figure 3). A single circumnutation cycle is determined by two subsequent extremes (maxima or minima). The extremes are detected on data smoothed using a moving average filter with a customizable filter size – the filter eliminates possible false extremes. Then, a discrete derivative is calculated to determine the slope of the curve. When the slope sign is changing – the extreme is detected, and when the slope changes from rising to falling, the extreme is the maximum (Figure 3; green dots) and otherwise – the minimum. We propose that in the geographic direction plane, the single circumnutation cycle is determined by two subsequent maximum northward bends of the organ (Figure 3; red dots). Figure 3 also shows that the regular and irregular patterns of circumnutation can be elaborated successfully by *CT*.

The following circumnutation parameters: period, length, rate, shape, and clockwise- and counter-clockwise directions are calculated by *CT* for every single circumnutation cycle.

•*period* (min) - the time that the organ apex needs to trace a single circumnutation cycle (time between two subsequent maximum northward bends of the organ).

•*length* (the length of the trajectory of circumnutation, mm) - the way that the organ apex covers during a single circumnutation cycle (between two subsequent maxima northward bends, Figure 3). This parameter corresponds to the term “amplitude” widely used in the literature.

•*rate* - the apex rate during a single circumnutation cycle (circumnutation length divided by period, mm min^−1^).

•*shape (shape coefficient)* - a figure that is drawn in a horizontal plane by the organ apex during a single circumnutation, usually a wider or narrower ellipse or a circle. The shape is depicted by the shape coefficient, which is expressed as a ratio of the length of the short axis to the length of the long axis of the ellipse; for a very narrow ellipse, it is close to 0.1, and 1 for a circular circumnutation. Determination of the algorithm for the shape coefficient of circumnutation is based on the ellipse fitting method described by Fitzgibbon A, Pilu M and Fisher RB [40]. Examples of narrow and wider circumnutations are shown in Figure 2.

•*angle* - the angle between the long axis of the ellipse and the line drawn by the direction of the geographic north–south (NS). The angle is determined with the arctangent of the long and short axis vector of an ellipse previously fitted into the circumnutation cycle. The N is a 0 degree, E −90 degree and W is a 90 degree (Figure 2C). The angle is not determined for the circular circumnutation. This parameter together with the shape coefficient could help in the study of a rosette-like trajectory, as reported by Baillaud L [41].

•*direction* - a clockwise (cw, clockwise, right-handed) or counter-clockwise (ccw, left-handed) direction of movement of the apex. Some circumnutations are indeterminate (marked as ?).

### Table of circumnutation parameters – data output

The circumnutation parameter values can be exported as a CSV (comma-separated values) file that can be opened in spreadsheet software (e.g. Microsoft Excel) as shown in Table 2.

**Table 2:** **Circumnutations parameters determined automatically; example of seedling number 14 on Additional file **1**: Video 1**

**Numero of cycle**	**Start of cycle**	**End of cycle**	**Middle of cycle**	**Period (min)**	**Length (mm)**	**Rate (mm min**^**-1**^**)**	**Shape**	**Angle (°)**	**Direction**
1	2013-07-30 18:35	2013-07-30 21:00	2013-07-30 19:47	145	17	0.12	0.13	-70	?
2	2013-07-30 21:00	2013-07-30 23:10	2013-07-30 22:05	130	32	0.25	0.14	-78	CW
3	2013-07-30 23:10	2013-07-31 01:45	2013-07-31 00:27	155	48	0.31	0.16	-78	CW
4	2013-07-31 01:45	2013-07-31 04:10	2013-07-31 02:57	145	40	0.28	0.19	-52	CCW
5	2013-07-31 04:10	2013-07-31 06:35	2013-07-31 05:22	145	39	0.27	0.28	-23	CCW
6	2013-07-31 06:35	2013-07-31 09:00	2013-07-31 07:47	145	45	0.31	0.50	17	CCW
7	2013-07-31 09:00	2013-07-31 11:30	2013-07-31 10:15	150	51	0.34	0.36	61	CCW
8	2013-07-31 11:30	2013-07-31 14:10	2013-07-31 12:50	160	56	0.35	0.66	86	CCW
9	2013-07-31 14:10	2013-07-31 17:10	2013-07-31 15:40	180	84	0.47	0.96	-15	CCW
10	2013-07-31 17:10	2013-07-31 20:10	2013-07-31 18:40	180	96	0.54	0.78	26	CCW
11	2013-07-31 20:10	2013-07-31 23:05	2013-07-31 21:37	175	103	0.59	0.73	45	CCW
12	2013-07-31 23:05	2013-08-01 02:05	2013-08-01 00:35	180	118	0.66	0.93	-87	CCW
13	2013-08-01 02:05	2013-08-01 05:15	2013-08-01 03:40	190	120	0.63	0.79	20	CCW
14	2013-08-01 05:15	2013-08-01 08:30	2013-08-01 06:52	195	104	0.54	0.62	35	CCW
15	2013-08-01 08:30	2013-08-01 11:40	2013-08-01 10:05	190	82	0.43	0.59	71	CCW
16	2013-08-01 11:40	2013-08-01 14:45	2013-08-01 13:12	185	64	0.35	0.83	79	CCW
17	2013-08-01 14:45	2013-08-01 18:00	2013-08-01 16:22	195	73	0.37	0.73	-88	CCW
18	2013-08-01 18:00	2013-08-01 21:10	2013-08-01 19:35	190	69	0.36	0.58	-84	CCW
19	2013-08-01 21:10	2013-08-02 00:20	2013-08-01 22:45	190	76	0.40	0.55	-52	CCW
20	2013-08-02 00:20	2013-08-02 04:00	2013-08-02 02:10	220	98	0.44	0.62	-41	CCW
21	2013-08-02 04:00	2013-08-02 08:00	2013-08-02 06:00	240	108	0.45	0.60	-4	CCW

### Trajectory of circumnutation – graph output

**Figure 4:**
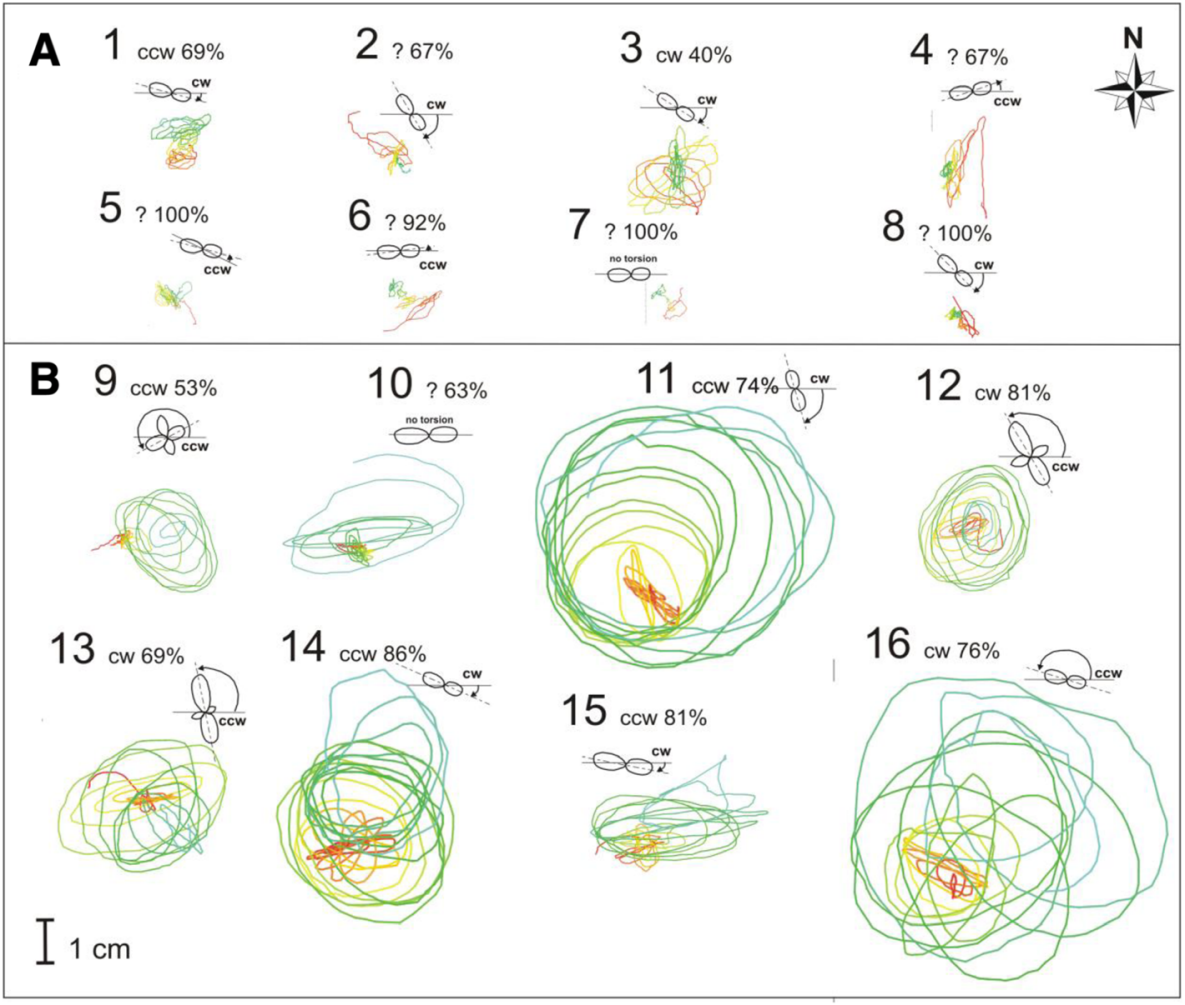
**The top view of the trajectory of hypocotyl circumnutation and cotyledon torsion of *Helianthus annuus *seedlings. A**. Distilled water. **B**. Nutrient solution. Cotyledon arrangement at the end of filming. Torsion in relation to beginning East–west cotyledon arrangement. All circumnutations during 63 hours of experiment. Numbers from 1 … 16 are numbers of seedlings in Additional file 1: Video 1.

The circumnutation trajectory can be seen in *CT* (Figure 4) and exported to a SVG (vector image) type file that can be opened in graphics software (e.g. Corel Draw). Simply, PrintScreen of trajectory of circumnutation is also possible.

An example of *CT* use for analysis of circumnutation of *Helianthus annuus* seedlings is presented below.

## Results and discussion

### Circumnutation parameters vs. morphology of seedlings of ***Helianthus annuus***

**Figure 5:**
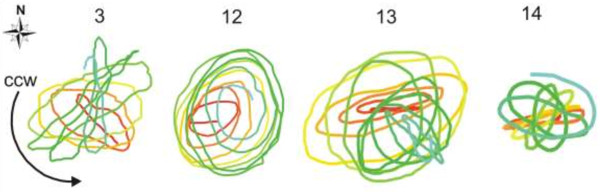
**The top view of the rosette-like trajectory of circumnutation of *Helianthus annuus *hypocotyls.** The seedlings (number 3, 12, 13, 14) are examples from Additional file 1: Video 1.

Dependence between plant morphology and the parameters of circumnutation was reported in *Avena*[7] and *Pisum*[32,33]. *Helianthus annuus* is a model plant for circumnutation and many studies have been carried out but no characteristics of circumnutations vs. seedlings morphology have been provided yet. Here, we have shown that circumnutations of seedlings growing in distilled water are different from those observed in plants growing in a nutrient solution (Additional file 1: Video 1). The parameters and trajectory of circumnutations determined using *CT* are shown in Table 1 and Figure 4. The distilled-water growing seedlings have a statistically significantly shorter hypocotyl (p < 0.00001) and length of circumnutation (p < 0.01) but a longer period (p < 0.01) than the nutrient-solution growing seedlings. The shape of distilled-water seedlings are slightly narrow ellipses (p < 0.05), and a dominating direction that is difficult to determine (73%). The nutrient-solution growing seedlings have wider ellipses, and *ccw* slightly dominated (42%) in relation to *cw* direction (34%). The long axis of the ellipses has the same angle arrangement on the geographical plane (−15°) in both groups and it is almost perpendicular to the axis of the cotyledon arrangement at the start of the experiment. Torsion of the hypocotyls was also observed and shown in Figure 4. The slowly growing distilled-water seedlings showed small torsion in relation to the faster growing nutrient-solution seedlings. The direction of torsion was usually counter to the dominating circumnutation direction as can be seen in seedlings no. 11, 12, 13, 14, 15, and 16 in Figure 4. Sometimes a rosette-like trajectory pattern was observed and its direction was usually ccw, as shown in Figure 5. The results presented above show the use of the *CT* software. The period and geometrical properties of subsequent circumnutation cycles are determined in irregularly and regularly circumnutating plants growing in different nutrient conditions. We expect that the analysis of circumnutation geometry will contribute to future studies of plant morphogenesis, including phyllotactic patterns and flower development [37,42].

## Materials and methods

### Experimental plants

*Helianthus annuus* L. seeds (PNOS, Ożarów Maz., Poland) were germinated on wet filter paper in a thermostated (25 ± 1°C) darkened chamber. Four-day (after 10 hours in light for hook opening) seedlings with 4.5 ± 0.5-cm long hypocotyls were cultivated hydroponically (eight plants per pot) in aerated distilled water and a nutrient solution (Additional file 1: Video 1). The nutrient solution contained 4 mM Ca(NO_3_)_2_ × 4H_2_O, 5 mM KNO_3_, 1 mM NH_4_H_2_PO_4_, 2 mM MgSO_4_ × 7H_2_O; microelements: 0.085 mM Fe(III)citrate, 0.046 mM H_3_BO_3_, 0.0009 mM MnCl_2_ × 4H_2_O, 0.0003 mM CuSO_4_ × 5H_2_O, 0.0008 mM ZnSO_4_ × 7H_2_O, 0.0001 mM H_2_MoO_4_ × 2H_2_O. The seedlings were set so that the cotyledons were arranged parallel to axis EW (Additional file 1: Video 1). The hydroponic culture was maintained for 63 hours under constant illumination, 40 μmol m^−2^ s^−1^ white light (Power Star HQT-T400 W/D OSRAM GmbH, Munich, Germany), at a temperature of 25 ± 1°C and relative humidity 50-70%.

### Circumnutation measurements – time-lapse video

For circumnutation measurements, time-lapse video recordings started at 18:00 p.m. on the fourth day and ended at 09:00 a.m. on the seventh day of seedling growth (Additional file 1: Video 1). A monochromatic camera (Mintron MTV-1368CD, Mintron Enterprise Co. Ltd, Taipei, Taiwan) was used to record the circumnutation trajectory of the hypocotyl apex. The camera parameters (focus, aperture, and exposure time) remained constant during the experiment. The plants were filmed from the top and the camera was oriented corresponding to geographical plane and thus the coordinates × and y were simultaneously east–west (EW) and north–south (NS) of the geographical direction, respectively. Time-lapse images were recorded one frame per 5 minute by Gotcha! Multicam software (Prescient System Inc., West Chester, PA, USA). The system was calibrated by filming the line with a millimetre scale at the level of the organ origin (ground level). The time-lapse images were digitized using *Circumnutation Tracker* and Microsoft Excel programs. Experimental points (coordinates ×, y of the stem apex on the horizontal plane) were determined at 5-min intervals.

### Statistical analysis

The results obtained are presented as the mean ± SE in each experimental group. The results were estimated for significance by a t-test at p < 0.05 and p < 0.01, p < 0.00001.

## Conclusions

We think that the *CT* software could be a useful tool for future research of circumnutation behaviour and may allow finding movement phenotypes [43]. The *CT* is an easy tool facilitating the circumnutation research, which could be helpful for plant physiology researchers and students. Therefore, the *CT* is available on http://circumnutation.umcs.lublin.pl. The user guide, explanation of installation, and samples are accessible. Nowadays, given the fast development of digital image recording [44,45], the time-lapse method of recording plant movement will develop rapidly; therefore, suitable software for time-lapse video analysis is required. Our software requires a minimal custom-made video input and can be adapted to different low-budget time-lapse imaging setups. In future, we will also work on fully automated harvesting of coordinates x y and 3D circumnutation-growth modelling.

### Availability and requirements

•Project name: *Circumnutation Tracker*

•Project home page: http://circumnutation.umcs.lublin.pl

•Operating system(s): Windows (XP, Vista, 7), Linux

•Programming language: C++

•Other requirements: Qt, libavcodec, blas, lapack, armadillo

*CT* is freely available from the authors' web pages and source code are freely available on request. *CT* can be used, modified and distributed freely as long as this publication and the original authors are acknowledged. If research projects benefited much from *CT*, this publication should be cited in arising papers.

## Abbreviations

CT: Circumnutation Tracker; cw: Clock-wise; ccw: Counterclock-wise.

## Competing interests

The authors declare that they have no competing interests.

## Authors’ contributions

MS wrote of this paper, designed the software architecture and methods. MŻ programming a software. EK and HD revised the final manuscript. All authors read and approved the final manuscript.

## Supplementary Material

Additional file 1: Video 1 A time-lapse video of circumnutation of *Helianthus annuus* seedlings growing in distilled water and nutrient solution. Click here for file
